# Visualization of ligand-induced transmembrane signaling in the full-length human insulin receptor

**DOI:** 10.1083/jcb.201711047

**Published:** 2018-05-07

**Authors:** Theresia Gutmann, Kelly H. Kim, Michal Grzybek, Thomas Walz, Ünal Coskun

**Affiliations:** 1 Paul Langerhans Institute Dresden, Helmholtz Zentrum München, University Hospital and Faculty of Medicine Carl Gustav Carus, Technische Universität Dresden, Dresden, Germany; 2 German Center for Diabetes Research, Neuherberg, Germany; 3 Laboratory of Molecular Electron Microscopy, The Rockefeller University, New York, NY

## Abstract

Insulin receptor (IR) signaling plays a critical role in the regulation of metabolism and growth in multicellular organisms. IRs are unique among receptor tyrosine kinases in that they exist exclusively as covalent (αβ)_2_ homodimers at the cell surface. Transmembrane signaling by the IR can therefore not be based on ligand-induced dimerization as such but must involve structural changes within the existing receptor dimer. In this study, using glycosylated full-length human IR reconstituted into lipid nanodiscs, we show by single-particle electron microscopy that insulin binding to the dimeric receptor converts its ectodomain from an inverted U-shaped conformation to a T-shaped conformation. This structural rearrangement of the ectodomain propagates to the transmembrane domains, which are well separated in the inactive conformation but come close together upon insulin binding, facilitating autophosphorylation of the cytoplasmic kinase domains.

## Abstract (precis)

Using single-particle electron microscopy of the human insulin receptor reconstituted into nanosdiscs, Gutmann et al. show that ligand binding induces a conformational rearrangement in the receptor ectodomain that results in the dimerization of the transmembrane domains and receptor activation.

## Graphical Abstract

**Figure:**
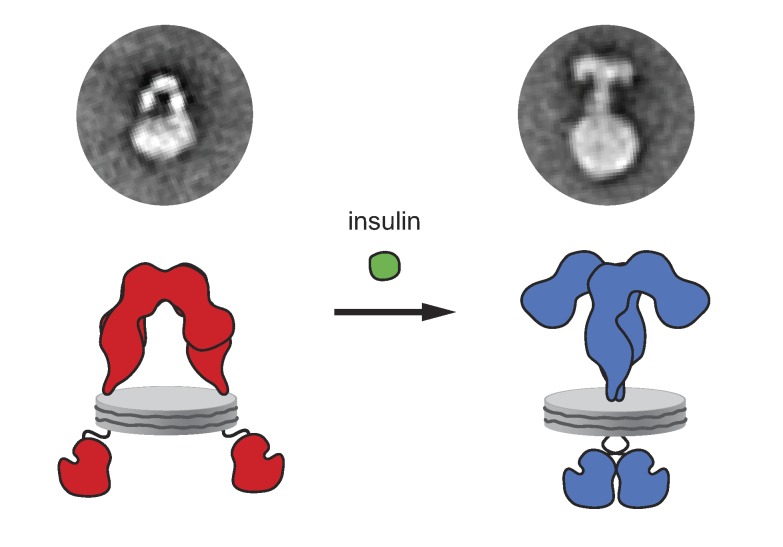

## Introduction

The insulin receptor (IR) is a ubiquitously expressed receptor tyrosine kinase (RTK) that is of fundamental importance for the regulation of glucose, protein, and lipid metabolism and growth (Saltiel and Kahn, 2001). Its dysfunction has been linked to severe pathologies including diabetes mellitus, cancer, and Alzheimer’s disease (Haeusler et al., 2018).

**Figure 1.**
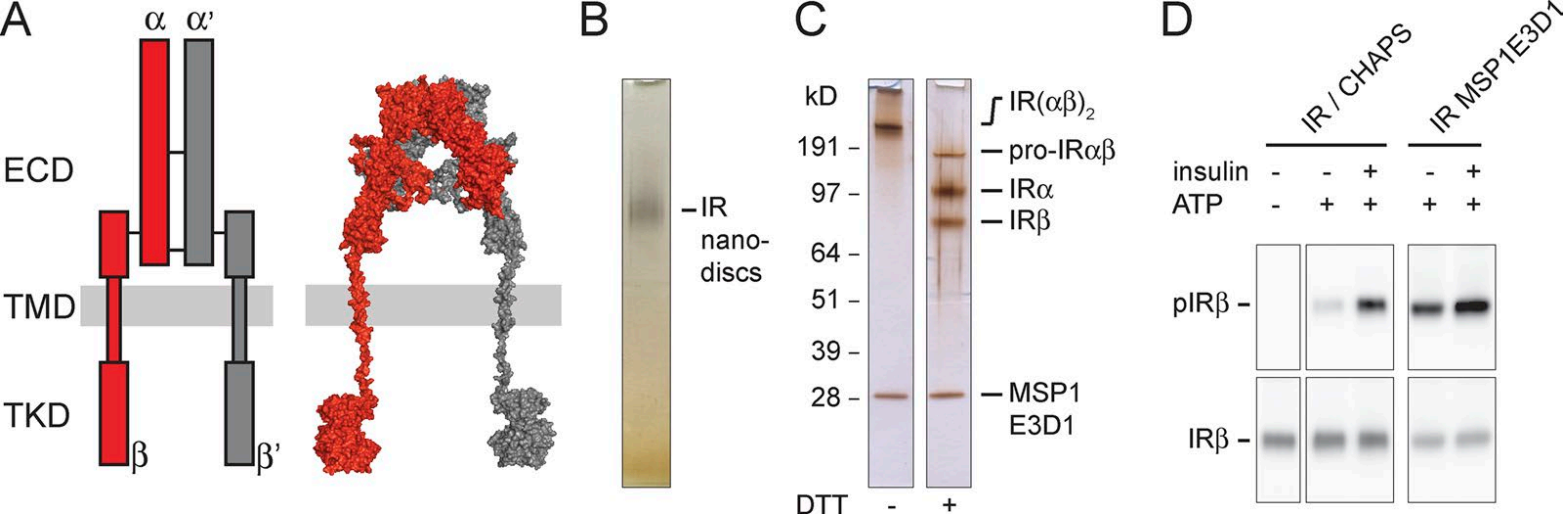
**IR reconstitution into nanodiscs and activity assay. (A)** Left: Schematic cartoon showing the ectodomain (ECD), transmembrane domain (TMD), and tyrosine kinase domain (TKD). Right: Structural model of the full-length human (αβ)_2_ IR assembled from available crystal structures of the tyrosine kinase (Hubbard et al., 1994) and extracellular domain (Croll et al., 2016) as well as the nuclear magnetic resonance solution structure of the transmembrane domain (Li et al., 2014). **(B)** Silver-stained native PAGE of glycosylated full-length human IR reconstituted into MSP1E3D1 nanodiscs. **(C)** SDS-PAGE of nanodisc-embedded IRs under nonreducing (−DTT) and reducing conditions (+DTT). **(D)** Activity assay showing that both CHAPS-solubilized and nanodisc-embedded IRs are autophosphorylated upon exposure to insulin. IRα, IR α subunit; IRβ, IR β subunit; IR(αβ)_2_, mature IR; pIRβ, phosphorylated IRβ; pro-IRαβ, unprocessed intracellular form of IR. Experimental details are provided in the In vitro phosphorylation assay and SDS-PAGE and Western blots sections in Materials and methods as well as Figs. S1 and S2.

In the canonical model of RTK signaling, receptor activation is mediated by ligand-induced dimerization and oligomerization (Ullrich and Schlessinger, 1990). However, a subset of RTKs can form noncovalent but inactive dimers before ligand binding (Maruyama, 2014; Freed et al., 2015). IRs are unique among RTKs in that they exclusively exist as covalently linked (αβ)_2_ homodimers at the cell surface (Fig. 1 A). Independent of their oligomeric state, all RTKs are activated by their cognate ligands, arguing that distinct allosteric changes within the dimeric receptor structure may contribute to receptor activation and signaling outcome (Maruyama, 2014; Freed et al., 2017).

Our structural understanding of IRs derives largely from crystallographic and nuclear magnetic resonance studies of receptor fragments (Hubbard et al., 1994; McKern et al., 2006; Menting et al., 2013; Li et al., 2014; Cabail et al., 2015; Croll et al., 2016), most notably those of an unliganded ectodomain (Croll et al., 2016) and a truncated ectodomain in complex with insulin (Menting et al., 2013). Attempts to image full-length IRs by EM yielded inconclusive results and did not reveal ligand-dependent structural changes (De Meyts and Whittaker, 2002), leaving the structural basis for transmembrane signaling to be elucidated (De Meyts, 2015; Tatulian, 2015).

To obtain insights into IR activation, we reconstituted recombinant full-length IR into lipid nanodiscs and visualized them by single-particle EM. In the absence of insulin, the IR ectodomain adopted the symmetric inverted U-shaped conformation described in previous studies of the ectodomain (Tulloch et al., 1999; McKern et al., 2006; Croll et al., 2016). Upon insulin binding, however, the receptor ectodomain converted into a T-shaped conformation that brought the transmembrane domains together, presumably facilitating autophosphorylation of the tyrosine kinase domains.

## Results and discussion

To elucidate the mechanism underlying transmembrane IR signaling, we produced recombinant full-length human (αβ)_2_ IRs (Fig. 1 A) in freestyle HEK293F cells, purified them in CHAPS to near-homogeneity by a single affinity chromatography step, and used membrane scaffold proteins (MSPs; Denisov et al., 2004; Schuler et al., 2013) to reconstitute them into lipid nanodiscs (Fig. 1, B and C; and Fig. S1). For reconstitution, we used a ternary lipid mixture consisting of phosphatidylcholine, sphingomyelin, and cholesterol at a ratio that forms homogeneous membranes in the liquid-disordered phase (Tumaneng et al., 2010). Assaying kinase activity upon exposure to insulin demonstrated increased autophosphorylation activity over basal levels for both the detergent-solubilized and the nanodisc-embedded IR (Figs. 1 D and S2), although the latter exhibited considerably higher background activity.

### Inactive IRs exhibit an inverted U-shaped ectodomain conformation and well-separated transmembrane domains

**Figure 2.**
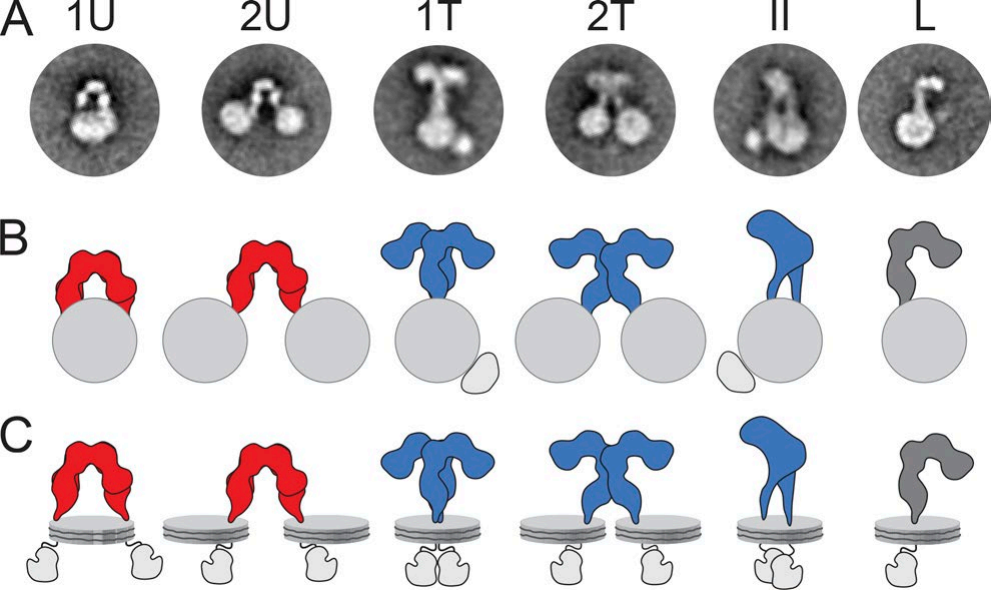
**Schematic illustration of the conformations assigned to IRs in nanodiscs. (A and B)** Representative class averages (A) and schematic drawings (B) of IRs in nanodiscs adopting different conformations: U-shaped dimer (ectodomain in red) in one (1U) or two nanodiscs (2U), T-shaped dimer (ectodomain in blue) in one (1T and II) or two nanodiscs (2U), and L-shaped monomer (ectodomain in dark gray) in one nanodisc (L). **(C)** Models of how the nanodisc-embedded IRs may look in solution. Because the tyrosine kinase domains are flexibly tethered to the transmembrane domains, their locations are somewhat speculative, especially in the 2T conformation. Note, however, that extra density in the class averages of the 1T and II conformations may represent tyrosine kinase domain dimers.

**Figure 3.**
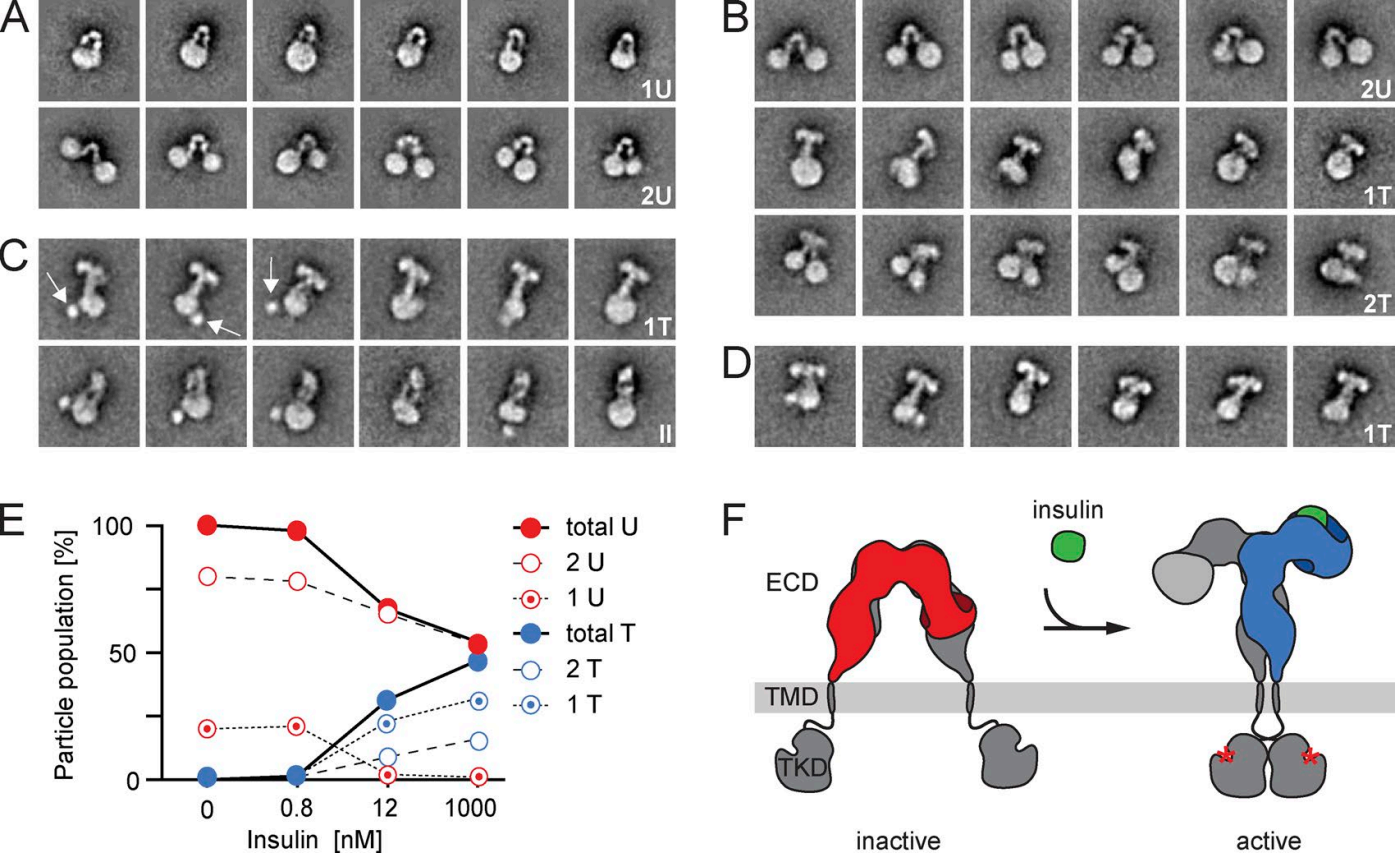
**Selected EM averages of IRs reconstituted into MSP1E3D1 nanodiscs and ligand-induced IR activation. (A)** In the absence of insulin, IRs reconstituted into one nanodisc (1U; top) or two nanodiscs (2U; bottom) and adopted a U-shaped conformation. **(B)** Upon insulin addition, all IRs in a single nanodisc adopted the T-shaped conformation (1T; middle), whereas IRs in two nanodiscs adopted either the U-shaped (top) or T-shaped conformation (2T; bottom). **(C)** In the presence of insulin, all IRs reconstituted into a single nanodisc. Most of them adopted a T-shaped conformation (top), whereas some showed an II-shaped conformation (II), likely representing a different view of IRs in the T-shaped conformation (bottom). The first three averages show a globular density that may represent dimerized tyrosine kinase domains (TKDs; arrows). **(D)** Upon insulin addition, all IRs showed a T-shaped conformation. The side length of individual panels is 47.7 nm. **(E)** Quantification of IR populations with a particular ectodomain (ECD) conformation at given insulin concentrations. **(F)** Cartoon illustrating the ligand (green) binding–induced conformational change in the ectodomain and its coupling to the transmembrane domains (TMDs) with concomitant activation of the tyrosine kinase domains by autophosphorylation (red asterisks). Our experimental data imply that binding of one ligand is sufficient to induce the transition of the IR ectodomain from the U-shaped to the T-shaped conformation.

Full-length IRs were first reconstituted with MSP1E3D1, an MSP variant that forms nanodiscs of ∼12 nm in diameter (Denisov et al., 2007), and the resulting structures were imaged by negative-stain EM. Although heterogeneous, many particles showed circular shapes representing nanodiscs, from which additional densities extended (Fig. S3 A). About 10,000 interactively selected particles were subjected to *K*-means classification into 200 classes (Fig. S3 B) and to iterative stable alignment and clustering (ISAC; Fig. S3 C; for classification details, see Table S1). *K*-means classification assigns every particle to one of the specified number of classes and can therefore be used to quantify particle populations (see numbers throughout the study). ISAC only uses a subset of the particles, namely those that fall into stable and reproducible classes. ISAC is thus not suitable to quantify particle populations, but it produces averages with very reliable and crisp features (for more details, see Yang et al., 2012). Some ISAC averages revealed an L-shaped density extending from a nanodisc (Fig. S3 C), presumably representing unprocessed monomeric IRs (see Fig. 2 for an interpretation of the 2D class averages in terms of the 3D structures they likely represent). Because monomeric IRs do not exist at the cell surface under physiological conditions (Hedo et al., 1983), they will not be discussed further. Many ISAC averages showed an inverted U-shaped density that either extended from a single nanodisc (20%; see the Image processing section of Materials and methods for estimation of particle populations; Fig. 3 A, top) or connected two nanodiscs (80%; Fig. 3 A, bottom). The width of the U-shaped density (∼8–11 nm) was consistent with the respective IR ectodomain dimension seen in a previous negative-stain EM study (Tulloch et al., 1999) and in crystal structures (McKern et al., 2006; Croll et al., 2016). The unliganded ectodomain extended 8.5–10 nm from the edge of the nanodiscs, which presumably is an underestimation of the total length of the ectodomain because the nanodisc density makes it impossible to identify the precise position where the protein emerges from the membrane. However, as most IR dimers reconstituted into two separate nanodiscs, the two transmembrane domains must generally be well separated from each other in unliganded receptors.

### Insulin binding results in structural rearrangements of the receptor that allow the transmembrane domains to come together

Ligand binding by IRs displays negative cooperativity. The first ligand binds with high affinity and is suggested to suffice for receptor activation under physiological ligand concentrations (Lee and Pilch, 1994). At concentrations of ∼1–100 nM, a second insulin molecule can bind with lower affinity but accelerates dissociation of the first ligand. At ligand concentrations >1 µM, three insulin molecules may occupy the receptor (De Meyts and Whittaker, 2002; Kiselyov et al., 2009).

To understand the consequences of insulin binding, IRs were first incubated with a saturating insulin concentration of 1 µM before they were reconstituted into nanodiscs. EM images of this IR preparation showed that all receptors were incorporated into a single nanodisc (Fig. 3 C). All averages showed a dramatic conformational change in the ectodomains from the U-shaped conformation predominantly to a T-shaped conformation (∼72%; Fig. 3 C, top). A smaller fraction showed II-shaped ectodomains (26%), presumably representing a different view of the T-shaped conformation (Fig. 3 C, bottom). Additional exposure to 1 µM insulin after reconstitution showed that all dimeric IR ectodomains adopted the T-shaped conformation (Fig. 3 D). Importantly, because all ligand-bound IR dimers were incorporated into a single nanodisc, the two transmembrane domains must be in close proximity and presumably interact with each other.

To confirm that the conformational change also occurs in membrane-embedded IRs, we used IRs reconstituted into nanodiscs in the absence of insulin (Fig. 3 A) and exposed them to 1 µM insulin. Averages still revealed IRs in the U-shaped conformation connecting two nanodiscs (Fig. 3 B, top), but these constituted a smaller fraction (∼53%). Strikingly, all IRs that were reconstituted into a single nanodisc appear to have undergone the conversion from the U-shaped to the T-shaped conformation upon insulin binding (Fig. 3 B, middle). The T-shaped receptors had a wingspan of ∼12.5–14.5 nm and extended ∼11–13.5 nm from the edge of the nanodiscs. In addition, averages now also showed T-shaped IRs extending from two nanodiscs (∼16%; Fig. 3 B, bottom). From these results, ligand binding to IRs reconstituted into two nanodiscs should convert their ectodomains into a T-shaped conformation, which, in turn, would exert a driving force to bring the transmembrane domains together. This force will tend to bring the two nanodiscs together, which was indeed seen in some of the averages (Fig. 3 B, bottom). Furthermore, nanodiscs connected by a T-shaped IR in general appeared to be closer to each other than those connected by a U-shaped IR (Fig. 3 B, compare top with bottom). The resulting juxtaposition of the two nanodiscs may only occasionally lead to fusion, allowing the transmembrane domains to interact, whereas in most cases, the two nanodiscs would likely remain separate, preventing the transmembrane domains from interacting.

To test whether the transition into the T-shaped conformation also occurs at nonsaturating ligand conditions, we exposed IRs embedded in nanodiscs to an insulin concentration of 12 nM as well as to the physiologically relevant and highly substoichiometric insulin concentration of 0.8 nM. The quantification of the receptors in different conformations (Fig. 3 E and Table S3) implied that one ligand is sufficient to induce the conversion of the IR ectodomain from the U- to the T-shaped conformation.

### In the absence of insulin, the transmembrane domains are well separated

Crystallographic studies established that in unliganded IRs, the membrane-proximal segments of the ectodomains are ∼12 nm apart (McKern et al., 2006; Croll et al., 2016). We therefore hypothesized that reconstituting unliganded IRs into smaller nanodiscs should increase the number of IRs that incorporated into two nanodiscs. To test this hypothesis, we repeated the above experiments with MSP1D1, an MSP that forms smaller nanodiscs of ∼10 nm diameter (Denisov et al., 2004). As expected, in the absence of insulin, all receptors adopted the U-shaped conformation. More importantly, with the smaller nanodiscs, the two transmembrane domains of the dimeric IRs incorporated exclusively into separate nanodiscs, strengthening the notion that the transmembrane domains do not interact with each other in unliganded receptors (Fig. S4 A). In the presence of insulin, however, even with the smaller nanodiscs, all IRs reconstituted into single nanodiscs (Fig. S4 C). In summary, all results were consistent with those obtained with IRs reconstituted into the larger nanodiscs (Fig. S4).

The T-shaped conformation of the activated IR described in this study resembles what has been seen in previous EM images of IRs in detergent and reconstituted into liposomes (Christiansen et al., 1991; Tranum-Jensen et al., 1994; Woldin et al., 1999). These studies, however, did not reveal conformational changes in the receptor upon exposure to insulin, whereas our data now provide direct structural evidence for a ligand-driven conformational change in the IR ectodomain and its importance for signal propagation across the membrane. In the absence of such data, mutually exclusive models have been proposed for IR activation (De Meyts and Whittaker, 2002; Ward et al., 2013; Kavran et al., 2014; Lee et al., 2014; De Meyts, 2015; Tatulian, 2015). In one model, IR transmembrane domains interact with each other in the inactive state, and insulin binding then pries them apart (Lee et al., 2014). In an opposing model proposed for the homologous insulin-like growth factor-1 receptor, the ectodomains keep the transmembrane domains separated in the inactive state. Ligand binding would then release this inhibition and allow the transmembrane domains to interact (Kavran et al., 2014). An inhibitory role of the ectodomain has also been suggested for inactive preformed dimers of the EGF receptor in which the unliganded ectodomain exerts steric constraints to prevent the association of the N-terminal segments of the transmembrane helices (Zhu et al., 2003; Arkhipov et al., 2013). In line with this model, tryptic removal of the IR ectodomain was shown to result in constitutively active kinase domains (Shoelson et al., 1988). Our results unambiguously support the latter model also for IR regulation (Fig. 3 F): insulin binding induced a large conformational change in the IR ectodomain, releasing the constraint on the transmembrane domains and concomitantly facilitating autophosphorylation of the kinase domains. Indeed, some averages of the receptor in the T-shaped conformation showed a strong globular density (arrows in Fig. 3 C) that was not seen in averages of the receptor in the U-shaped conformation, suggesting that the tyrosine kinase domains in these averages may be more restricted in their localization and dimerized.

Given the importance of the rearrangements in the IR transmembrane domains, we envision that the lipid environment will have an effect on receptor activation and regulation, a notion that can be tested further using the nanodisc system. Our results also provide novel structural insights that answer longstanding questions concerning the mechanism of how insulin activates the receptor, thus improving our understanding of the receptor and providing new perspectives for pharmacological intervention strategies.

## Materials and methods

### Expression and purification of full-length IRs

cDNA for human IR isoform A was cloned into a pTT6 vector as described previously (Gerl et al., 2014), resulting in a construct consisting of the human IR isoform A followed by a C-terminal human rhinovirus 3C protease cleavage site and a tandem affinity purification tag.

The receptor was produced in suspension-adapted FreeStyle HEK293F cells (RRID: CVCL_D603; Thermo Fisher Scientific) that were grown at 37°C in protein-free FreeStyle 293 expression medium (Thermo Fisher Scientific) supplemented with 1× penicillin/streptavidin at 90 rpm and 8% CO_2_. Cells at 2 × 10^6^ cells/ml in antibiotics-free medium were transiently transfected with 1 mg/liter endotoxin-free plasmid DNA (precomplexed with polythelenimine at a 1:8 [wt/wt] ratio; Gerl et al., 2014). The cells were maintained for 64 h at 31°C, 8% CO_2_, and 90 rpm. After harvesting the cells by centrifugation at 1,000 *g* for 10 min, the cell pellet from 1 liter of cell culture was resuspended in 100 ml ice-cold detergent-free HBS (50 mM Hepes, pH 7.5, and 150 mM NaCl, supplemented with 8 mM Pefabloc, 10 µM E64, and two tablets of complete protease inhibitor cocktail [Roche]). Resuspended cells were disrupted using a tip sonicator (Ultrasonic Homogenizer Sonopuls HD 3100 with an SH70 G horn and MS 73 microtip [3 mm]; Bandelin Electronic) in the absence of detergent by four to six sonication cycles (1 s pulse and 1 s pause for 30 s total followed by 1 min pause at 17% amplitude). Protein purification was performed at 4°C as described previously for EGF receptor (Coskun et al., 2011), except that the pH of all purification buffers was 7.5 and that after the washing step with 20% (vol/vol) glycerol-containing running buffer, the column was equilibrated with HBS supplemented with 1% (wt/vol) CHAPS and 8% (vol/vol) glycerol for overnight incubation with human rhinovirus 3C protease and protein elution.

IR concentration was estimated using a molar extinction coefficient of 377,585 M^−1^ cm^−1^ as calculated by ProtParam (ExPASy), assuming 42 of the total 94 cysteines are forming disulfide bonds in the dimeric receptor (Croll et al., 2016). The typical yield of purified IR was ∼0.5 mg from 1 liter of cell culture.

For quality controls, analytical size-exclusion chromatography was performed using a Superose 6 3.2/300 column (GE Healthcare) with IR elution buffer at a flow rate of 0.05 ml/min at room temperature followed by SDS-PAGE of the collected fractions and silver staining. Purified IR was immediately reconstituted into nanodiscs.

### Purification of MSP1E3D1 and MSP1D1

Plasmids encoding MSP pMSP1E3D1 (20066) and pMSP1D1 (20061) were purchased from Addgene. Both MSP variants were produced in *E. coli* BL21Gold(DE) (Agilent Technologies) and purified as described previously (Inagaki et al., 2012) with the exceptions that prepacked His-Trap HP columns (GE Healthcare) were used for immobilized metal ion affinity chromatography and that an additional wash step with cholate was introduced (50 mM Tris, pH 7.4, 200 mM NaCl, and 32 mM cholate). The His tag was not removed.

### Reconstitution of IRs into nanodiscs

1,2-dioleoyl-*sn*-glycero-3-phosphocholine (DOPC), *N*-stearoyl-d-erythro-sphingosyl-phosphorylcholine (SM), and cholesterol were purchased from Avanti Polar Lipids. All lipid stocks were quantified on a regular basis to correct for solvent evaporation by a phosphate assay for phospholipids (Rouser et al., 1966) or an Amplex red cholesterol assay kit (Invitrogen) and were controlled for their stability by thin-layer chromatography. DOPC, SM, and cholesterol were mixed at a ratio of 80:15:5 mol%, dried under a stream of nitrogen, and left under vacuum overnight to remove residual solvent. To better solubilize the lipid mixture containing cholesterol and sphingomyelin and to improve reproducibility, the dried film was first used to form liposomes by addition of reconstitution buffer (20 mM Hepes, pH 7.5, and 100 mM NaCl) with vigorous shaking at 54°C followed by 10 freeze-thaw cycles and 21 extrusions through a 100-nm pore-size polycarbonate filter. The resulting unilamellar liposomes were then solubilized by addition of cholate (Alfa Aesar) to a final concentration of 18 mM and subsequent sonication in a water bath.

IRs were reconstituted into nanodiscs in the presence or absence of human insulin (I2643; Sigma-Aldrich). For reconstitutions in the presence of insulin, 0.3 µM IR in 50 mM Hepes, pH 7.5, 150 mM NaCl, 1% (wt/vol) CHAPS, and 8% (vol/vol) glycerol was incubated with 1 µM insulin for 1 h on ice. Before usage, insulin was dissolved to a concentration of 180 µM in 5 mM HCl. Insulin was added to the IR reconstitution mix to a final concentration of 1 µM. Even though the pH of the solution did not change, the same amount of 5 mM HCl was added to the unliganded receptor to rule out potential effects of HCl on IR. For nanodisc formation, 0.3 nmol liganded or unliganded IR was incubated with 200 nmol cholate-solubilized lipids and 10 nmol MSP1E3D1 or MSP1D1 for 30 min in the presence of 13.2 mM CHAPS at 25°C with gentle shaking at 300 rpm. The reconstitution mix was centrifuged at 20,000 *g* for 20 min at 4°C, and the supernatant was extensively dialyzed against at least a 1,000× excess of 20 mM Hepes, pH 7.5, and 100 mM NaCl for 26 h at 4°C with three buffer exchanges followed by a fourth exchange to 25 mM Hepes, pH 7.5, and 150 mM NaCl for 18 h. Dialysis was performed in Spectra/Por 7 standard regenerated cellulose pretreated dialysis tubings (Spectrum Labs) with a molecular weight cutoff of 10 kD (for the first 18 h) and 50 kD (until the end of the dialysis). Because of the aggregation propensity of nonreconstituted MSPs, aggregates were removed by centrifugation at 150,000 *g* for 45 min at 4°C. The supernatant was snap-frozen in liquid nitrogen and stored at −80°C. IR-containing nanodiscs were separated from empty nanodiscs by size-exclusion chromatography using a Superose 6 10/300 GL column (GE Healthcare) with 25 mM Hepes, pH 7.5, and 150 mM NaCl, and only the peak fraction was collected.

For EM experiments of IR reconstituted into nanodiscs with MSP1E3D1, the peak fraction was split into four aliquots. One aliquot was imaged directly by negative-stain EM, and the other aliquots were incubated with varying amounts of insulin (final concentrations of 0.8 nM, 12 nM, or 1 µM) for 1 h at 4°C before imaging. For IR reconstituted into nanodiscs with MSP1D1, the peak fraction was split into halves. One half was imaged directly by negative-stain EM, whereas the other half was incubated with 1 µM insulin (final concentration) for 1 h at 4°C before imaging.

The concentration of IR in nanodiscs was estimated by UV spectroscopy at 280 nm with a molar extinction coefficient of 438,030 M^−1^ cm^−1^ for samples reconstituted with MSP1E3D1 and 420,445 M^−1^ cm^−1^ for samples reconstituted with MSP1D1 as calculated with ProtParam (ExPASy) assuming that 42 of the 94 cysteines formed disulfide bonds.

### In vitro phosphorylation assay

A saturating insulin concentration (200 nM) was added to purified CHAPS-solubilized IRs or nanodisc-embedded IRs and allowed to bind for 1 h on ice. Phosphorylation was performed with 0.25 mM ATP in 25 mM Hepes, pH 7.5, 150 mM NaCl, 10 mM MgCl_2_, and 1 mM MnCl_2_ in a final volume of 25 µl and incubated for 12 min at 25°C. For solubilized IRs, the CHAPS concentration was kept at the critical micellar concentration of 0.6% (wt/vol). The reaction was stopped by addition of SDS sample buffer supplemented with 2.5 mM EDTA. The reaction mixtures were subjected to SDS-PAGE, and IR tyrosine kinase domain phosphorylation was assessed by Western blotting.

### SDS-PAGE and Western blots

Electrophoresis was performed with precast 4–12% Bis-Tris gels with MOPS SDS running buffer or 3–8% Tris-acetate gels or NativePAGE 3–12% Bis-Tris gels (Invitrogen) using the corresponding commercial running buffers (Invitrogen). Proteins were stained with Coomassie blue or silver stain or were transferred to polyvinylidene difluoride membranes for immunodetection. Membranes were blocked overnight with 5% (wt/vol) nonfat dry milk powder in TBST (0.1% [vol/vol] Tween-20 in 20 mM Tris, pH 7.4, and 150 mM NaCl) supplemented with phosphatase inhibitors (1 µM NaVO_4_ and 20 µM β-glycerophosphate). Membranes were probed with antibodies against phospho-IRβ (Tyr-1150/1151; 1:1,000; 3024; RRID, AB_331253; Cell Signaling Technology) or against total IRβ C terminus (1:2,000; 3020; RRID, AB_2249166; Cell Signaling Technology). The secondary antibodies goat anti–rabbit IgG (H+L; 170-6515; RRID, AB_11125142; Bio-Rad Laboratories) or goat anti–mouse IgG (H+L; 170-6516; RRID, AB_11125547; Bio-Rad Laboratories) conjugated to horseradish peroxidase were used at a 1:10,000 dilution, and detection was performed using SuperSignal West Femto maximum-sensitivity substrate electrochemiluminescence substrate (Thermo Fisher Scientific) with a charge-coupled device imager (Imager 600; GE Healthcare).

### Specimen preparation and EM

An aliquot (3.5 µl) of full-length IR reconstituted into nanodiscs was adsorbed to a glow-discharged 200-mesh copper grid covered with a thin carbon-coated plastic film. After adsorbing the sample for 1 min, the grid was blotted with a filter paper and washed twice with buffer (25 mM Hepes, pH 7.5, and 150 mM NaCl), once with deionized water, and once with a 0.75% (wt/vol) uranyl formate solution, blotting the grid with filter paper between each drop, and then it was stained for 30 s with the uranyl formate solution. After blotting the grid again with filter paper, the remaining solution was removed by vacuum aspiration (see Ohi et al. [2004] for further details). Images were collected using an XR16L-ActiveVu charge-coupled device camera (AMT) on a Philips CM10 electron microscope (FEI) operated at an acceleration voltage of 100 kV. The calibrated magnification was 41,513× (nominal magnification of 52,000×), yielding a pixel size of 2.65 Å at the specimen level. The defocus was set to –1.5 µm.

### Image processing

Images of nanodisc-embedded IRs showed a very heterogeneous particle population. Therefore, for further image processing, particle selection focused on particles that showed one nanodisc with an additional density extending from them or two nanodiscs connected by a density. For each sample, ∼10,000 particles (the exact numbers of images and selected particles are summarized in Table S1) were manually selected using the e2boxer.py command of the EMAN2 software package (Tang et al., 2007) and windowed into 180 × 180–pixel images. After image normalization and particle centering, the particle images were classified into 200 groups using *K*-means classification procedures implemented in SPIDER (Frank et al., 1996). For the ISAC algorithm (Yang et al., 2012) implemented in SPARX software (Hohn et al., 2007), the particle images were reduced to 76 × 76 pixels and subjected to several ISAC generations specifying 50 images per group and a pixel error threshold of 0.7 (the number of ISAC generations and resulting number of averages for each sample are summarized in Table S1).

To estimate the approximate number of receptors adopting a particular conformation, we used the *K*-means classification into 200 groups and visually assigned the averages to show (a) a U-shaped IR reconstituted into one nanodisc (1U), (b) a U-shaped IR reconstituted into two nanodiscs (2U), (c) a T-shaped IR reconstituted into one nanodisc (1T), (d) a T-shaped IR reconstituted into two nanodiscs (2T), (e) a II-shaped IR (II; we only observed IRs in this conformation associated with one nanodisc), and (f) an L-shaped IR (L; we only observed L-shaped IRs associated with one nanodisc, consistent with the assumption that these particles represent IR monomers). For each set of averages, the particles in the classes assigned to one of these six classes were summed up and are listed in Table S2. Many of the averages could not be unambiguously assigned to one of the six classes. These classes (X) were considered uninterpretable and were excluded from the quantification. The assignments of the classes for each experimental condition are denoted in Fig. S3 C and dataviewer content showing the 200 SPIDER class averages. In addition, the particles showing the L-shaped nonphysiological monomeric IR were not considered when calculating the percentage of dimeric receptors in each conformation (1U, 2U, 1T, 2T, or II) as listed in Table S3.

### Online supplemental material

Fig. S1 illustrates the biochemical analyses of purified IR and IR reconstitution into nanodiscs. Fig. S2 shows autophosphorylation assays for IRs in detergent and in nanodiscs. Fig. S3 presents EM analysis of IR reconstituted into large nanodiscs with MSP1E3D1 in the absence of insulin, showing an EM image and the class averages obtained by ISAC and *K*-means classification. Fig. S4 shows the conformation of IR reconstituted into small nanodiscs with MSP1D1 under different insulin exposure conditions. Table S1 provides statistics for EM image collection and ISAC classification. Table S2 provides the numbers of particles assigned to specific IR conformations, and Table S3 provides the corresponding percentages of the dimer population. 

## Supplementary Material

Supplemental Materials (PDF)
