# Supplementary material for: Visualization of ligand-induced transmembrane signaling in the full-length human insulin receptor
Source: J Cell Biol. 2018 May 7;217(5):1643–9. doi: 10.1083/jcb.201711047 (PMC5940312; doi:10.1083/jcb.201711047)
Supplement: Supplemental Materials (PDF) [file JCB_201711047_sm.pdf]

Supplemental material

Gutmann et al., https://doi.org/10.1083/jcb.201711047

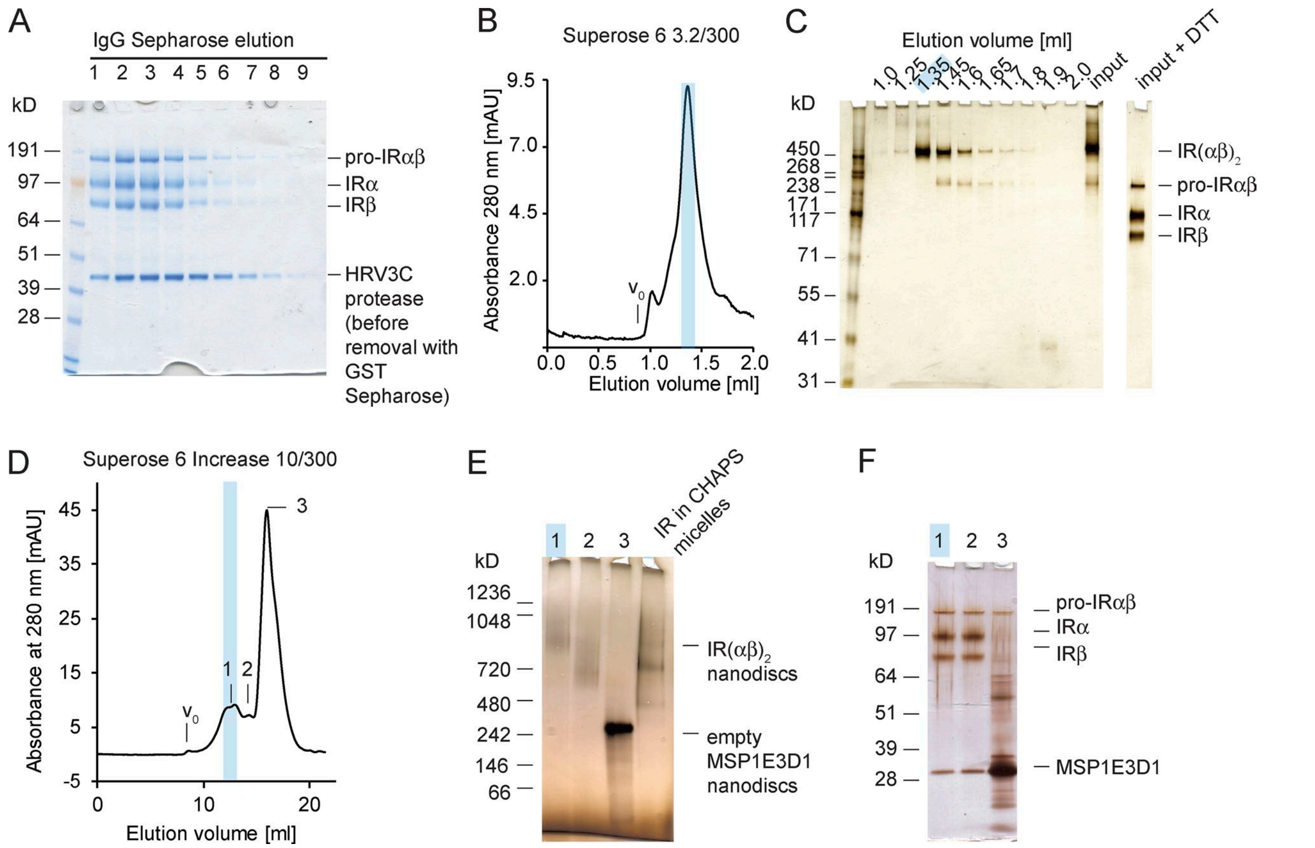

Figure S1. **Purification of full-length IRs and reconstitution into nanodiscs. (A)** Coomassie blue–stained SDS-PAGE gel showing fractions of IR elution from IgG Sepharose after 3C protease cleavage. The mature IR dimer (IR(αβ)$_2$) was reduced in the presence of DTT and separated into its α and β subunits (IRα and IRβ). Pro-IRαβ denotes the intracellular receptor in its pro form. IR is furin-cleaved in the trans-Golgi, and the pro form thus does not exist on the cell surface under physiological conditions (Hedo et al., 1983). **(B)** Superose 6 3.2/300 chromatogram of the IR shows the homogeneity of the preparation. The void volume is indicated (v_0). **(C)** Eluted fractions were analyzed by nonreducing SDS-PAGE and silver staining. The prominent band at 460 kD confirms the dimeric assembly of the IR. **(D)** Size-exclusion chromatogram of IR in MSP1E3D1 nanodiscs eluting from a Superose 6 Increase 10/300 column. **(E)** Native PAGE of the fractions indicated in D. **(F)** Reducing SDS-PAGE of the fractions indicated in D with subsequent silver staining.

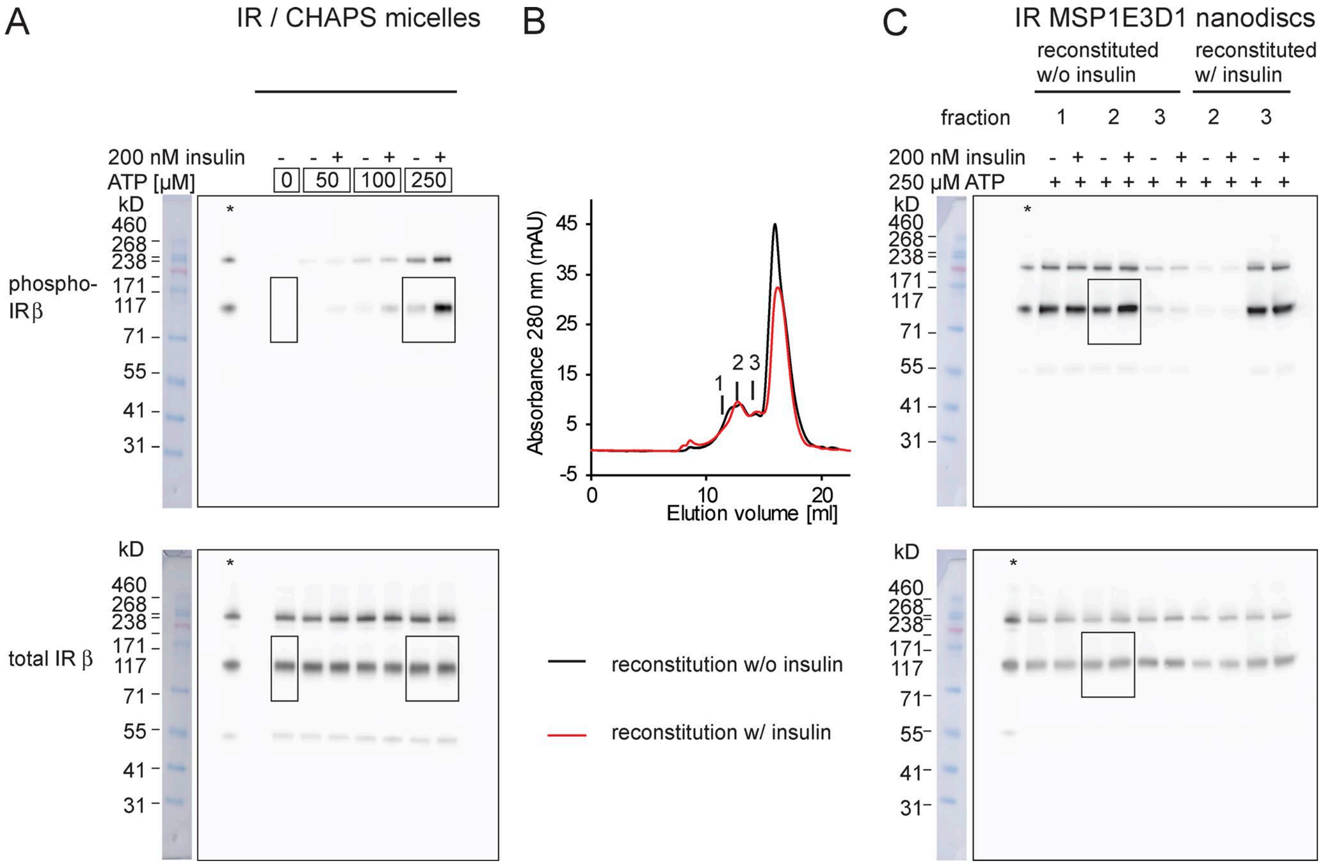

Figure S2. **Activity assay of purified IRs. (A–C)** Autophosphorylation was assessed with a phosphospecific anti-IRβ antibody (top), whereas the total protein amount was assessed by reprobing the same blots with antibody directed against the C terminus of IRβ (bottom). **(A)** Activation of CHAPS-solubilized IRs in the absence or presence of an insulin concentration of 200 nM and increasing concentrations of ATP. The increase in phosphorylated IRβ in the presence of insulin shows that the IRs are functional. The top band corresponds with the pro form, which is not discussed any further. **(B)** IRs reconstituted into MSP1E3D1 nanodiscs in the absence (black line) or presence (red line) of insulin were run over a Superose 6 10/300 Increase size-exclusion chromatography column. **(C)** The fractions marked in B were tested for insulin-induced phosphorylation. Only the fraction corresponding to nanodisc-embedded dimeric IRs reveals an increase in phosphorylation upon insulin addition. IRs that were reconstituted into nanodiscs in the presence of insulin did not show a further increase in phosphorylation when additional insulin was added after reconstitution. The results displayed in A and C are derived from the same experiment and were processed in parallel. Asterisks indicate loading controls of phosphorylated IRs (the same amount of the same sample was loaded on each gel).

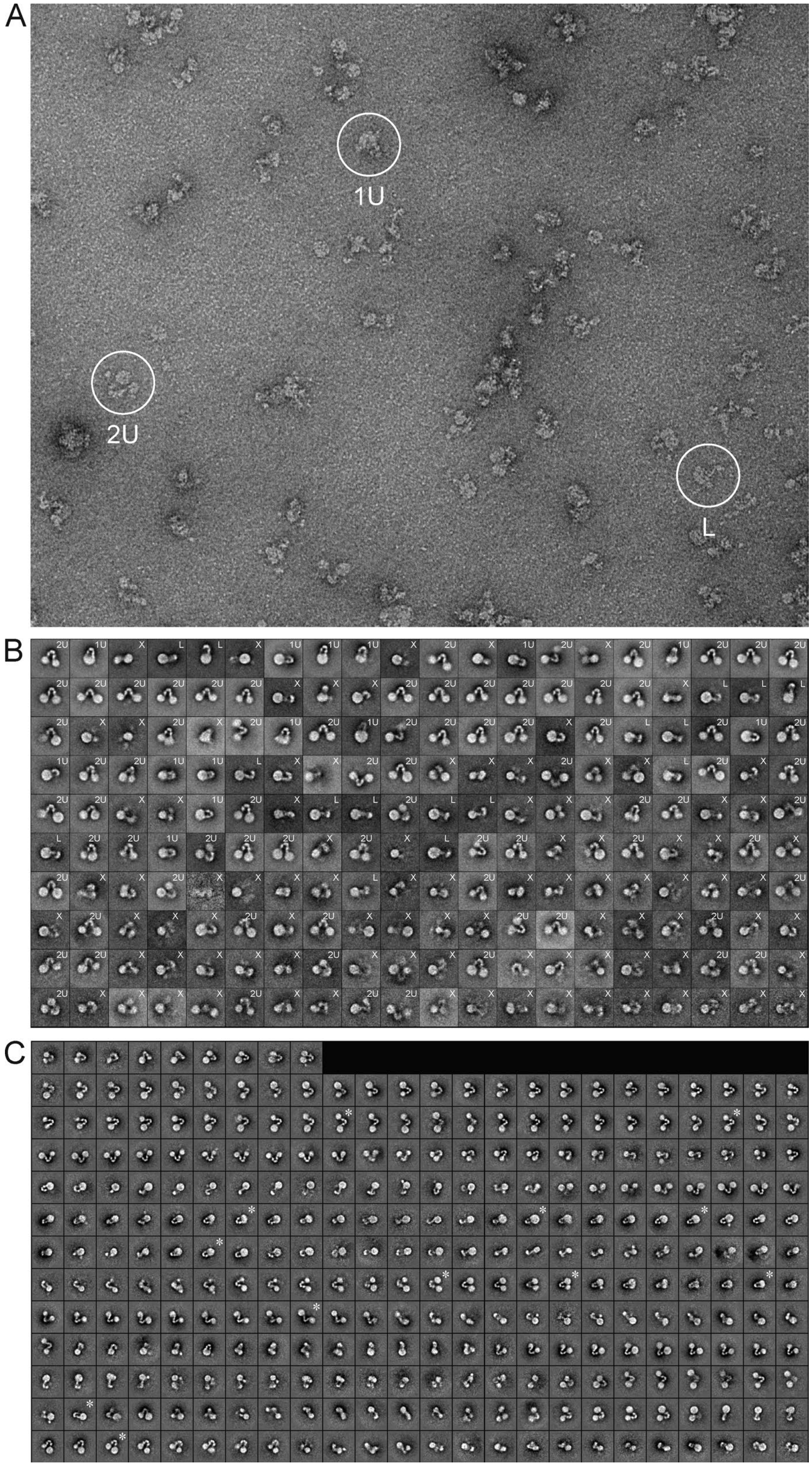

Figure S3. **EM analysis of IR reconstituted into large nanodiscs with MSP1E3D1 in the absence of insulin. (A)** Selected area of a raw negative-stain EM image. Some representative particles are circled. **(B)** The 200 class averages obtained from *K*-means classification of 13,800 particles. The class averages were visually assigned to show a U-shaped IR in one nanodisc (1U), a U-shaped IR in two nanodiscs (2U), an L-shaped IR (L), or none of these (X). **(C)** The 297 averages resulting from eight ISAC generations. The averages shown in Fig. 3 A are marked with asterisks. The side length of individual averages in B and C is 47.7 nm.

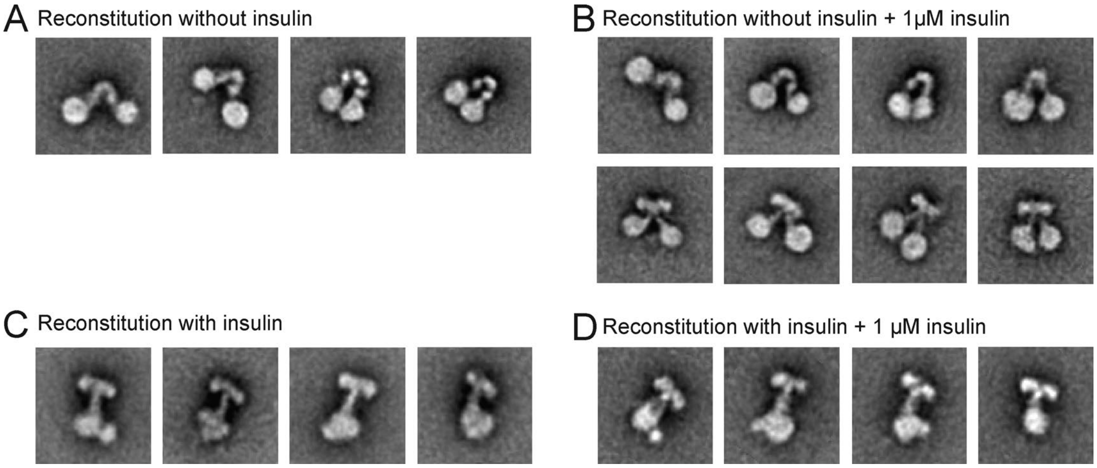

Figure S4. **Conformation of IRs reconstituted into small nanodiscs with MSP1D1 under different insulin exposure conditions. (A)** In the absence of insulin, all IRs reconstituted into two nanodiscs and adopted a U-shaped conformation. **(B)** Upon addition of 1 µM insulin, some IRs remained in the U-shaped conformation (top), but many converted into a T-shaped conformation while remaining in two nanodiscs (bottom). A small fraction of IRs was found in the T-shaped conformation in a single nanodisc (not depicted). **(C)** In the presence of insulin, IRs reconstituted into a single nanodisc, and the vast majority of them adopted a T-shaped conformation. **(D)** Upon addition of 1 µM insulin, almost all IRs showed a T-shaped conformation. The side length of individual panels is 47.7 nm.

Table S1. **Image collection and ISAC classification statistics.**

**Sample**	**Number of images**	**Number of particles**	**Number of ISAC generations**	**Number of ISAC averages**	**Number of particles assigned**	**Percent particles assigned**
						%
**IR reconstituted into MSP1E3D1 in the absence or presence of 1 µM insulin**						
Rec – Ins; no insulin added	200	13,800	8	297	12,103	88
Rec – Ins; +0.8 nM insulin	200	10,634	7	215	8,431	79
Rec – Ins; +12 nM insulin	250	10,858	7	230	9,267	85
Rec – Ins; +1 µM insulin	300	14,041	9	299	11,935	85
Rec + Ins; no insulin added	100	14,820	9	307	13,540	91
Rec + Ins; +1 µM insulin	100	10,619	5	202	8,364	79
**IR reconstituted into MSP1D1 in the absence or presence of 1 µM insulin**						
Rec – Ins; no insulin added	200	11,493	9	241	9,687	84
Rec – Ins; +1 µM insulin	400	13,304	13	286	11,900	89
Rec + Ins; no insulin added	100	10,239	3	166	7,258	71
Rec + Ins; +1 µM insulin	100	5,303	6	112	4,926	93

Rec – Ins, absence of insulin; Rec + Ins, presence of insulin.

Table S2. **Number of particles assigned to represent IRs in specified ECD conformations.**

Sample	Total number of particles	Number of particles assigned to specified classes						
		**2U**	**1U**	**2T**	**1T**	**II**	**L**	**X**
**IR reconstituted into MSP1E3D1 in the absence or presence of 1 µM insulin**								
Rec – Ins; no insulin added	13,800	5,655	1,416	0	0	0	1,345	5,384 (39.0%)
Rec – Ins; +0.8 nM insulin	10,634	4,472	1,212	0	44	0	496	4,372 (41.1%)
Rec – Ins; +12 nM insulin	10,858	3,171	87	452	1,099	0	1,077	4,972 (45.8%)
Rec – Ins; +1 µM insulin	14,041	2,859	0	864	1,713	0	1,364	7,241 (51.6%)
Rec + Ins; no insulin added	14,820	0	96	0	2,962	1,066	2,956	7,740 (52.2%)
Rec + Ins; +1 µM insulin	10,619	0	0	0	3,085	0	570	6,964 (65.6%)
**IR reconstituted into MSP1D1 in the absence or presence of 1 µM insulin**								
Rec – Ins; no insulin added	11,493	4,930	0	0	0	0	616	5,947 (51.7%)
Rec – Ins; +1 µM insulin	13,304	4,149	0	2,897	285	0	686	5,287 (39.7%)
Rec + Ins; no insulin added	10,239	84	162	20	3,136	0	543	6,294 (61.5%)
Rec + Ins; +1 µM insulin	5,303	17	135	0	2,226	0	452	2,473 (46.6%)

Rec – Ins, absence of insulin; Rec + Ins, presence of insulin.

Table S3. **Percentage of dimeric IR population in specified classes**

Sample	Number of particles assigned to specified classes						
	**2U**	**1U**	**Total U**	**2T**	**1T**	**Total T**	**II**
	%	%	%	%	%	%	%
**IR reconstituted into MSP1E3D1 in the absence or presence of 1 µM insulin**							
Rec – Ins; no insulin added	80.00	20.00	100	0	0	0	0
Rec – Ins; +0.8 nM insulin	78.1	21.10	99.20	0	0.80	0.80	0
Rec – Ins; +12 nM insulin	65.9	1.80	67.70	9.40	22.90	32.30	0
Rec – Ins; +1 µM insulin	52.6	0	52.60	15.90	31.50	47.40	0
Rec + Ins; no insulin added	0	2.30	2.30	0	71.80	71.8 (97.7[a])	25.90
Rec + Ins; +1 µM insulin	0	0	0	0	100	100	0
**IR reconstituted into MSP1D1 in the absence or presence of 1 mM insulin**							
Rec – Ins; no insulin added	100	0	100	0	0	0	0
Rec – Ins; +1 µM insulin	56.6	0	56.60	39.50	3.90	43.40	0
Rec + Ins; no insulin added	2.50	4.70	7.20	0.60	92.20	92.80	0
Rec + Ins; +1 µM insulin	0.7	5.70	6.40	0	93.60	93.60	0

Rec – Ins, absence of insulin; Rec + Ins, presence of insulin.
[a]Value if the II-shaped particles are included in the sum of T-shaped particles.

Reference

Hedo, J.A., C.R. Kahn, M. Hayashi, K.M. Yamada, and M. Kasuga. 1983. Biosynthesis and glycosylation of the insulin receptor. Evidence for a single polypeptide precursor of the two major subunits. *J. Biol. Chem.* 258:10020–10026.
